# Melanotic Neuroectodermal Tumor of Infancy (MNTI) and Pineal Anlage Tumor (PAT) Harbor A Medulloblastoma Signature by DNA Methylation Profiling

**DOI:** 10.3390/cancers13040706

**Published:** 2021-02-09

**Authors:** Oscar Lopez-Nunez, Rita Alaggio, Ivy John, Andrea Ciolfi, Lucia Pedace, Angela Mastronuzzi, Francesca Gianno, Felice Giangaspero, Sabrina Rossi, Vittoria Donofrio, Giuseppe Cinalli, Lea F. Surrey, Marco Tartaglia, Franco Locatelli, Evelina Miele

**Affiliations:** 1 Division of Pathology and Laboratory Medicine, Cincinnati Children’s Hospital Medical Center, Cincinnati, OH 45229, USA; oscar.lopeznunez@cchmc.org; 2 Pathology Unit, Bambino Gesù Children’s Hospital, IRCCS, 00165 Rome, Italy; sabrina2.rossi@opbg.net; 3 Department of Pathology and Laboratory Medicine, University of Pittsburgh Medical Center, Pittsburgh, PA 15213, USA; johni@upmc.edu; 4 Genetics and Rare Diseases Research Division, Bambino Gesù Children’s Hospital, IRCCS, 00165 Rome, Italy; andrea.ciolfi@opbg.net (A.C.); marco.tartaglia@opbg.net (M.T.); 5 Department of Pediatric Onco-Hematology and Cell and Gene Therapy, Bambino Gesù Children’s Hospital, IRCCS, 00165 Rome, Italy; lucia.pedace@opbg.net (L.P.); angela.mastronuzzi@opbg.net (A.M.); franco.locatelli@opbg.net (F.L.); 6 Radiologic, Oncologic and Anatomo-Pathological Sciences Department, Sapienza University, 00161 Rome, Italy; francesca.gianno@uniroma1.it (F.G.); felice.giangaspero@uniroma1.it (F.G.); 7 IRCCS Neuromed, Pozzilli, 86077 Isernia, Italy; 8 Pathology Unit, Azienda Ospedaliera Santobono-Pausilipon, 80122 Naples, Italy; v.donofrio@santobonopausilipon.it (V.D.); g.cinalli@santobonopausilipon.it (G.C.); 9 Department of Pathology and Laboratory Medicine, Perelman School of Medicine at the University of Pennsylvania, Philadelphia, PA 19104, USA; surreylf@email.chop.edu; 10 Department of Pediatrics, Sapienza University of Rome, 00161 Rome, Italy

**Keywords:** melanotic neuroectodermal tumor, melanotic progonoma, medulloblastoma, pineal anlage tumor, DNA methylation, copy number variation

## Abstract

**Simple Summary:**

Melanotic neuroectodermal tumor of infancy (MNTI) is a rare tumor of uncertain origin, morphologically overlapping other rare neoplasms such as pineal anlage tumor (PAT) and a subset of medulloblastomas (i.e., melanotic medulloblastoma). Despite the similarities with MNTI, their possible histogenetic relationship has been traditionally disregarded based on their aggressive behavior and dismal prognosis. The aim of this study was to further characterize the molecular features of MNTI and PAT based on DNA-methylation and copy number variation profiling analysis. We found that MNTI shares a methylation profile with group 3 high-risk medulloblastoma, and potentially with PAT, suggesting a common histogenesis. Most MNTIs in our series lacked copy number variation alterations, whereas their presence in the one PAT deserves further study in larger cohorts to better determine their impact in prognosis and biologic behavior.

**Abstract:**

MNTI is a rare tumor of indeterminate histogenesis and molecular signature. We performed methylation and copy number variation (CNV) profiles in patients with MNTI (*n* = 7) and PAT (*n* = 1) compared to the methylation brain tumor classifier v11b4 (BT-C) and the medulloblastoma (MB) classifier group 3/4 v1.0 (MB3/4-C). The patients’ mean age was 8 months (range: 4–48). The BT-C classified five MNTIs and one PAT (relapse) as class family MB-G3/G4, subclass group 3 (score: >0.9). The remaining two MNTIs and PAT (primary) were classified as class family plexus tumor, subclass pediatric (scores: >0.45). The MB3/4-C classified all MNTIs as high-risk MB-G3, Subtype II (score: >0.45). The primary PAT was classified as subtype III (score: 0.99) and its relapse as subtype II/III. MNTI and PAT clustered close to MB-G3. CNV analysis showed multiple rearrangements in one PAT and two MNTIs. The median follow-up was 54 months (four MNTIs in remission, one PAT died). In conclusion, we demonstrated that MNTI shares a homogenous methylation profile with MB-G3, and possibly with PAT. The role of a multipotent progenitor cell (i.e., early cranial neural crest cell) in their histogenesis and the influence of the anatomical site, tumor microenvironment, and other cytogenetic events in their divergent biologic behavior deserve further investigation.

## 1. Introduction

Since its original description in 1918 by Krompecher et al., under the name of “congenital melanocarcinoma of the alveolar process” [1], the histogenesis of melanotic neuroectodermal tumor of infancy (MNTI) has been disputed, as evidenced by different names attributed to this entity over the past century. Halpert and Patzer proposed a histogenetic relationship of these tumors with the ciliary process and retinal nuclear layers by introducing the term “retinal anlage tumor” [2], a notion later contested by Stowens, who instead coined the term “melanotic progonoma” to emphasize the absence of an anatomical precursor (and its putative origin from misplacement of tissue due to fetal atavism) while highlighting the characteristic presence of pigmented cells [3]. Finally, the production of vanillylmandelic acid (VMA) observed in one such case led Borello and Gorlin to propose a neuroectodermal origin, coining the current terminology of MNTI [4]. Although MNTI is currently considered a neurocristic tumor [5,6], only very few studies have attempted to further address its underlying molecular profile, most of them on a case report basis [7,8,9].

Interestingly, MNTI overlaps with the rare pineal anlage tumor (PAT), which is regarded as a variant of pineoblastoma [10]. It also shows morphologic overlap with melanotic medulloblastoma, which was proposed as a possible variant of MNTI [11]. However, this theory is debatable given the usual aggressive course of melanotic medulloblastomas in contrast to the generally benign behavior of MNTI [12]. Recently, Ebel et al. reported a rapidly growing skull tumor in a male infant with a methylation profile of group 3 medulloblastoma despite showing conventional MNTI morphology, raising further questions regarding the underlying pathobiology of these neoplasms [13]. Intriguingly, we recently came across a PAT showing a similar methylation profile. On this ground, we sought to explore the DNA methylation and copy number variation profiling in a series of seven MNTIs and one PAT to better delineate their histogenesis.

## 2. Results

### 2.1. Clinical Findings

The index case was a PAT, diagnosed in a 12-month-old boy. This patient presented with a local relapse, occurring 2 months after the original diagnosis. Additionally, seven MNTIs were identified in various anatomic locations, including the maxilla (*n* = 4), hard palate (*n* = 1), neck (*n* = 1), and pretesticular region (*n* = 1). The median age at diagnosis was 8 months (range: 4–48 months) with a male-to-female ratio of 6:2. All MNTI patients with available clinical information (*n* = 4) were in clinical remission. In addition, the PAT patient died of disease 16 months after the original diagnosis following a multistage surgical resection approach (https://doi.org/10.1007/978-3-030-16006-7 (accessed on 5 February 2021)). The median follow-up was 54 months. Follow-up information was lost in the remaining cases.

### 2.2. Morphologic and Immunophenotypic Findings

**Figure 1:**
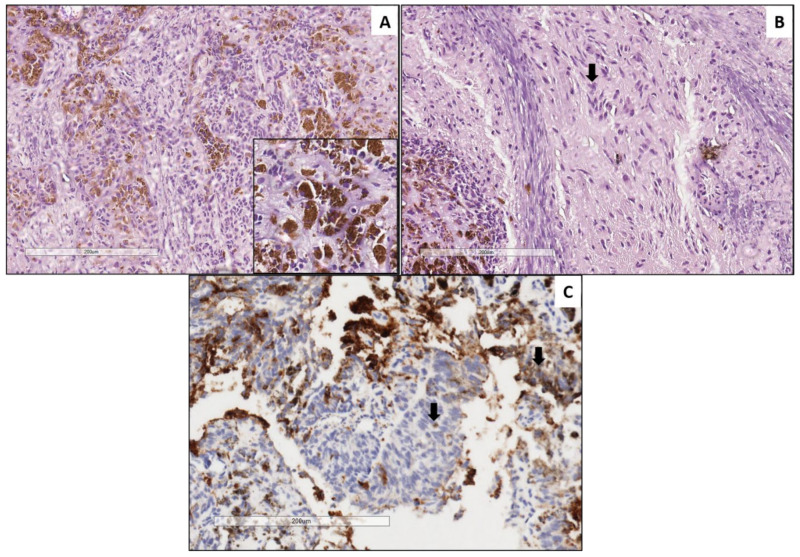
Pineal anlage tumor (PAT, primary specimen). Neuroepithelial component characterized by pineoblastoma-like nests of small round blue cells with hyperchromatic nuclei admixed with tubular structures composed of epithelioid cells containing melanin pigment (inset) (**A**). Ganglion cells characterized by round nuclei and abundant eosinophilic cytoplasm (arrow) (**B**). Positive immunoreactivity for synaptophysin in a cytoplasmic granular pattern (arrow) (**C**).

**Figure 2:**
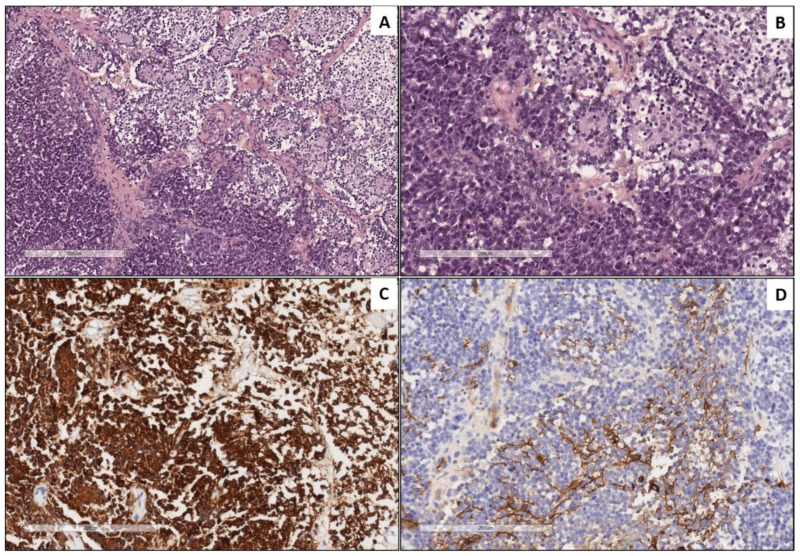
Pineal anlage tumor (relapse specimen). Neuroepithelial component characterized by solid areas of small round blue cells with hyperchromatic nuclei and high N:C ratio (left side) associated to Homer-Wright rosettes (right side) ((**A**) and (**B**), respectively). Synaptophysin staining neuroepithelial cells (**C**). Scattered neoplastic glial cells positive for glial fibrillary acidic protein (**D**).

**Figure 3:**
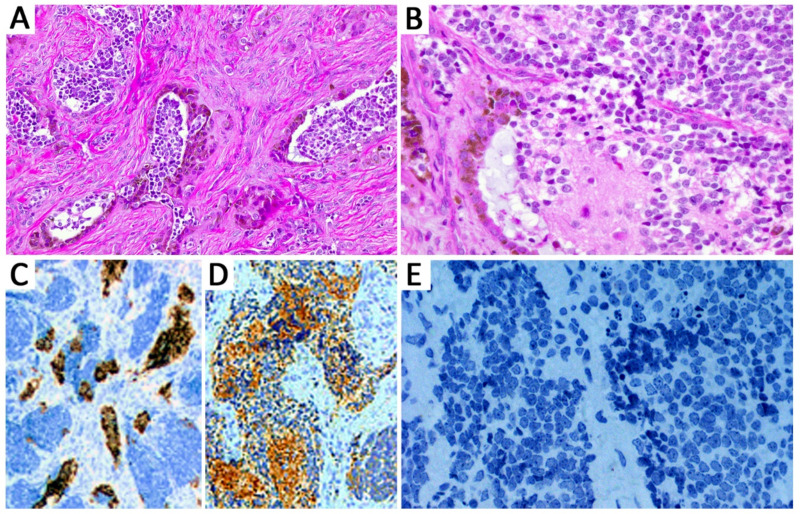
Melanotic neuroectodermal tumor of infancy (MNTI). Tumors showed a biphasic pattern composed of small round, neuroblast-like cells forming clusters/nests alternating with larger cells with more abundant cytoplasm and variable melanotic pigment in a dense fibrous stroma (**A**,**B**). All MNTIs had a large cell component consistently positive for HMB-45 (**C**). The small, neuroblast-like cell component was strongly reactive for synaptophysin (**D**), while Phox2b was constantly negative in all components (**E**).

The index case, a PAT, demonstrated a variably pigmented epithelial component similar to MNTIs (Figure 1), admixed with areas featuring ectomesenchymal differentiation (fibrous tissue and multiple foci of cartilage), whereas the recurrence was predominantly composed of primitive embryonal cells, with areas of neuronal differentiation resembling neuroblastic areas of MNTIs (Figure 2). All MNTI exhibited a characteristic biphasic pattern composed of small round, neuroblast-like cells forming clusters/nests alternating with larger cells with more abundant cytoplasm and variable melanotic pigment in a dense fibrous stroma (Figure 3A,B). The mitotic activity was overall low (average: 0–2 mitoses per 10 high-power fields), and no significant atypia or tumoral necrosis was identified. All MNTI had a large cell component consistently positive for HMB-45 (Figure 3C) and cytokeratin AE1/AE3 with variable synaptophysin expression. The small, neuroblast-like cell component was strongly reactive for NSE and synaptophysin (Figure 3D), while Phox2b was constantly negative in all components (Figure 3E).

### 2.3. Methylation Profiling

The methylation data of the PAT were first categorized using the brain tumor classifier v11b4 (https://www.molecularneuropathology.org/mnp/classifier/2 (accessed on 5 February 2021)) [14], which also generated a copy number variation (CNV) plot. The primary tumor clustered in the class family plexus tumor, subclass pediatric (PLEX, PED B), with suboptimal raw and calibrated scores (>0.45), while its relapse was classified as group 3 medulloblastoma (MB G3) with optimal scores (Table 1).

However, it has been reported that tumors that cannot be correctly classified (with a score > 0.95) are often classified as PLEX, PED B while there is no clear connection to this tumor group. Classifier developers suggest that a low score for PLEX PED B in an unexpected setting should therefore be considered with caution [15].

No significant methylation scores were reached in the sarcoma classifier v12.2 (https://www.molecularneuropathology.org/mnp/classifier/9 (accessed on 5 February 2021)). When exploiting the “Medulloblastoma classifier group 3/4 v1.0”, which takes into account the new consensus on the second-generation molecular subgrouping of medulloblastoma [16], the PAT was classified as subtype III (calibrated score 0.99) and its relapse as subtype II/III (Table 1).

**Figure 4:**
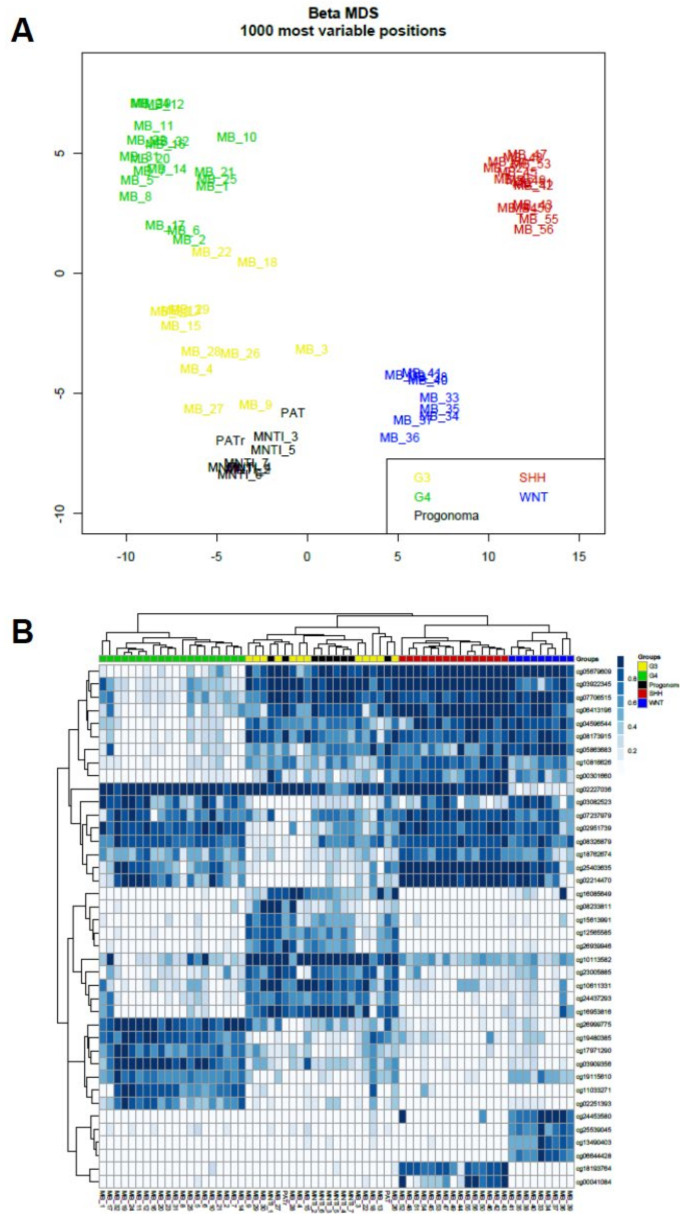
Multidimensional scaling (MS) analysis performed on the 1000 most variable probes of the whole-genome DNA methylation data shows a close similarity between (MNTI/PAT) and MBG3. Color legend of the MDS plot as follows: Progonoma (MNTI/PAT, black); WNT, Wingless MB (blue); SHH Sonic Hedgehog (red); G3, Group 3 MB (yellow); G4, Group 4 MB (green) (**A**). Hierarchical clustering and heatmap of beta values relative to the 39 high-quality CpG islands better discriminating MB subgroup in the Hovestadt set (Hovestadt et al.). The heatmap shows normalized methylation levels in MNTI/PAT samples and MB samples. Clusters were obtained by means of Ward’s minimum variance method using the Euclidean distance. Color legend: Progonoma (MNTI/PAT, black); WNT, Wingless MB (blue); SHH-A, Sonic Hedgehog MB (red); G3, Group 3 MB (yellow); G4, Group 4 MB (green) (**B**).

**Figure 5:**
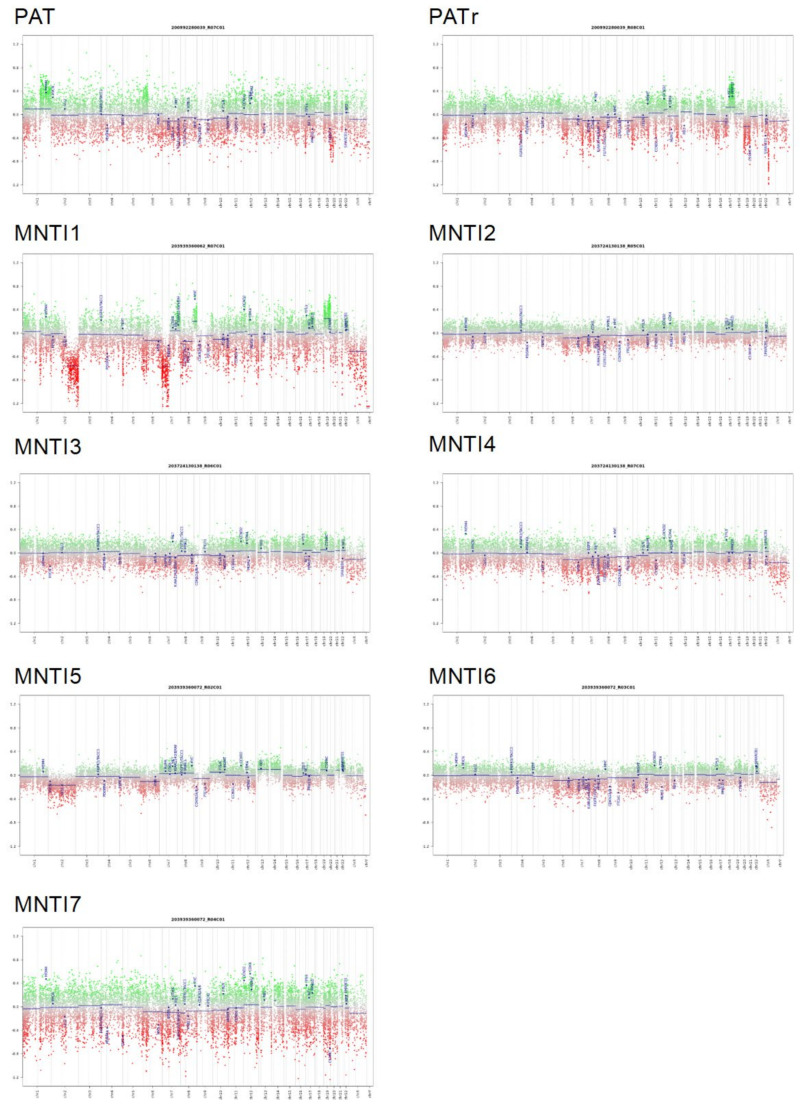
Copy number variation (CNV) profile analysis of the indicated PAT/MNTI. Depiction of structural rearrangements involving autosomes and X/Y chromosome. Gains/amplifications represent positive (green) and losses represent negative (red) deviations from the baseline. Twenty-nine tumor relevant genomic regions are highlighted.

**Figure 6:**
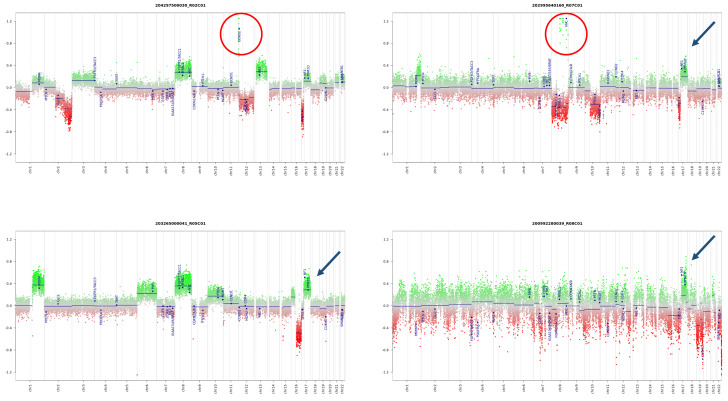
CNV of four different MBG3, subtype II analyzed at Ospedale Pediatrico Bambino Gesù. The CNV analysis shows recurrent gene and chromosomal alterations (e.g., *MYC* amplification [red circles] and Chr 17q gain (blue arrows)).

Subsequently, the global methylation profile of MNTI (*n* = 7) was analyzed. Five tumors clustered in the class MB G3 with optimal scores (Figure S1 and Supplementary information). The two remaining MNTIs clustered in the PLEX, PED B methylation class, with suboptimal calibrated scores (>0.45), while the maximum raw classification scores for both samples were reached in the MB G3 methylation class (Figure S1 and Supplementary information). In the “Medulloblastoma classifier group 3/4 v1.0”, all MNTI were classified as high-risk medulloblastoma G3 tumors, subtype II with a score > 0.45 (Table 1 and Supplementary information). In light of these results, we further analyzed and compared MNTI and PAT methylation data with those derived from a cohort of 36 medulloblastoma patients, diagnosed and treated at the Ospedale Pediatrico Bambino Gesù (OPBG Rome) [17]. All MNTI samples and PATs displayed a global methylation profile close to those of MB G3, as evidenced by both multidimensional scaling analysis performed on the 1000 most variable islands in the cohort (Figure 4A) and hierarchical clustering analysis (Figure 4B and Figure S2) performed on the reduced 48 CpG islands signature used to characterize the medulloblastoma subgroups [18]. Copy number variation (CNV) analysis showed an almost flat profile in five MNTIs (cases 2–6), multiple whole chromosomal gains and losses (including *MYC* amplification) in one MNTI (case 1) and PAT (both primary and recurrent specimen), and a highly variable profile in MNTI7 (Figure 5), similar to those observed in MB G3 in the control cohort (Figure 6).

**Figure 7:**
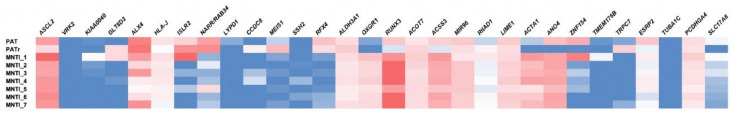
Heatmap depicting the top 30 differentially regulated genes among MNTI/PAT samples vs. MBG3. Color scale: Hypermethylated genes are reported in red, while hypomethylated genes are in blue.

To gain insight into the functional significance of the methylation data, we then analyzed the differentially methylated regions (DMRs) between MNTI/PAT and Group 3 medulloblastoma (Figure 7, Tables S1–S3). Functional enrichment analysis showed few statistically significant pathways (Table S4) (*p* values < 0.001, none with False Discovery Rate (FDR) < 0.01), supporting the hypothesis of common histogenesis and developmental programs.

## 3. Discussion

MNTI is a rare neoplasm of putative neural crest origin, preferentially arising in craniofacial sites. The histogenesis of MNTI remains elusive, with divergent theories (odontogenic, primordial germ cell, and a neural crest origin) being hypothesized over the years, leading to inconsistent—and confusing—nomenclature in the literature [6,19]. Although MNTIs are currently considered neoplasms of neurocristic origin [5,6], only very few studies have attempted to further address their underlying molecular and epigenetic profile [7,8,9]. This has been partly due to their rarity, with only about 500 cases reported until now [19,20,21]. As such, our understanding of their biology and prognosis remains elusive. Although most appear indolent despite their primitive “embryonal” morphology, there are reports of rare metastasizing cases with poor outcomes [6,22,23].

The histologic diagnosis of MNTI is relatively straightforward and rarely represents a diagnostic challenge, provided it arises from the usual anatomic sites and adequate tissue material is available for examination. In our series, most MNTI originated from the head and neck region except in case 7, which presented as a right paratesticular mass. These findings are consistent with other reported cases as most MNTI involve the head and neck [20], with a subset occurring in the paratesticular region [24,25,26] and other unusual locations [19]. Histologically, all MNTI had biphasic morphology characterized by small primitive neuroblast-like cells and larger peripheral epithelioid cells containing melanin granules confirmed by immunohistochemistry. Remarkably, the possibility of metastatic neuroblastoma was excluded in all cases by negative imaging studies and negative expression of Phox2b, which is considered a highly sensitive and specific immunohistochemical marker for tumors of neuroblastic origin, whereas it was reported negative in MNTI [27]. This immunostain can be used as a diagnostic aid in patients presenting with increased urinary levels of VMA, particularly in cases with limited biopsy material containing a predominantly neuroblastic-like morphology and expression of neuronal markers (i.e., synaptophysin, chromogranin A, PGP9.5, CD56).

The MNTI morphology overlaps with other more aggressive entities, including PAT [1,28,29] and medulloblastoma with melanotic differentiation [11,30]. Both lesions reveal a primitive neuroblastic-like component and epithelioid cells containing melanin pigment and were considered in the past as intracranial variants of MNTI [31,32]. As a matter of fact, PATs were initially named based on their similarities with MNTI (historically designated as retinal anlage tumor). However, their highly aggressive biologic behavior (in contrast to MNTI) has led to a critical reappraisal of these tumors, which are now considered variants of medulloblastoma and pineoblastoma, respectively [33]. Interestingly, PAT is characterized by a distinctive morphologic spectrum that includes a combination of neuroectodermal and heterologous ectomesenchymal components without endodermal elements [34]. This histological heterogeneity has raised the question of whether they truly represent a variant of classic pineoblastoma or a separate entity [33]. Interestingly, a recent study revealed different molecular subgroups among pineoblastomas, exhibiting substantial molecular heterogeneity based on DNA-methylation profiling. In particular, two cases diagnosed as PAT were classified by DNA-methylation profiling in the “pineoblastoma, *MYC-FOXR2*-activated group” [35]. In addition, Uro-Coste et al. reported a posterior fossa tumor (morphologically reminiscent of PAT) composed of undifferentiated monotonous cells, brisk mitotic activity, and foci of chondroid and rhabdomyoblastic differentiation exhibiting molecular features of an embryonal tumor with multilayered rosettes (ETMR)-like with *DICER1*-mutation in an infant [36].

Medulloblastomas are currently divided into four histologically distinct entities (classic, desmoplastic/nodular, extensive nodularity, and large cell/anaplastic) and four genetically defined subgroups (WNT-activated, SHH- activated TP53-wildtype, SHH-activated TP53-mutant, and non-WNT/non-SHH) [31]. Non-WNT/non-SHH medulloblastomas are consistently separated into group 3 (MB G3) and 4 by clustering large methylation array datasets. MB G3 are mainly known for their aggressive clinical behavior, with the worst and shortest survival among infants [37]. Notably, medulloblastoma with melanotic differentiation is an unusual tumor whose cell of origin is still debated (despite being considered a variant of medulloblastoma). Interestingly, a neural crest origin has been proposed based on the ultrastructure and proteomic features of its pigment resembling oculocutaneous melanin, including premelanosomes (rather than neuromelanin) [38]. Recently, Rajeshwari et al. reported a WNT-activated medulloblastoma with melanotic differentiation and speculated about the potential contribution of neural crest cell progenitors into their pathobiology [39].

This study found a compelling relationship between MNTI, PAT, and MB G3 based on their overlapping methylation profiles. Our results are in line with a recent report by Ebel et al., who described a 4-month-old boy with an MNTI arising from the skull and clustering in the class family of MB G3 by methylation studies [13]. In our series, 5/7 MNTI clustered in the class family MB G3 with an optimal score (>0.9), supporting the findings reported by Ebel et al. [13], as well as our index case (PAT, score 0.99). The remaining two MNTI cases clustered in the class family PLEX, PED B, with suboptimal scores (>0.45). However, when analyzed with the “Medulloblastoma classifier group 3/4 v1.0”, all seven MNTIs classified as high-risk MB G3 tumors, subtype II, with a score >0.45. Furthermore, compared with methylation data from a cohort of 36 medulloblastoma patients [17], all MNTIs and PATs displayed a global methylation profile very close to those of MB G3.

Interestingly, among the top 30 differentially methylated genes shown in Figure 3, 10 are reported to be implicated in development, morphogenesis or neural patterning (*VRK2*, *ALK4*, *ISLR2*, *NARR/RAB34*, *LYPD1*, *MEIS1*, *SSH2*, *RFX4*, *MIR96*, *TUBA1C*) (Table 2). Of note, three of these (*ALK4*, *MEIS1*, and *MIR96*), together with another six (*ASCL2*, *CCDC8*, *RUNX3*, *MIR96*, *ACTA1*, *ESRP2*), have been associated with WNT/β-catenin signaling as direct target genes or indirect regulators. It is known that WNT/β-catenin signaling has a widespread influence in cellular differentiation, tumor initiation, and progression [40,41]. This suggests there is a closer relationship between WNT/β-catenin signaling and non-WNT medulloblastomas (i.e., group 3 and group 4 medulloblastoma) [42].

Our series proposes that MNTI, PAT, and MB G3 could be part of a spectrum of neoplasms primarily determined by the influence of neural crest-derived stem cells, an appealing hypothesis deserving further investigation. Our findings support the longstanding notion of a histogenetic relationship between these three entities based on their molecular profiles and highlights a risk for potential diagnostic pitfalls. Nonetheless, the divergent benign behavior of MNTIs in contrast to PATs and MB G3 deserves further investigation. Possibly, additional cytogenetic events and site-related growth factors could play a role in these tumors’ biologic behavior. In fact, several subtypes (high risk vs. low risk) of MB G3 have recently emerged based on methylation profiles and frequency of *MYC* amplification, among others [43]. Interestingly, most MNTIs in our series (5/7; 71%) showed no CNVs in clear contrast to our PAT and MB G3 in the control cohort (Figure S4). We speculate that the absence of CNV may account, at least in part, for the indolent clinical behavior typically seen in most MNTIs. This discrepancy could also be related to other factors, including differences in oncogene activation, anatomical location, and tumor microenvironment. The divergence in biological behavior and treatment response based upon location has been suggested previously in other tumors [44]. Furthermore, most MNTIs arise from extracranial sites, potentially allowing for earlier identification and complete surgical resection [45] in comparison to PATs and medulloblastomas. Besides, most cancers coexist with other cells and cytokines, collectively known as the tumor microenvironment [46]. Some of these cellular and chemical mediators appear overexpressed in medulloblastomas, including molecules such as IL-8 and TGF-beta, as well as tumor-infiltrating T lymphocytes [47], that may differ from PATs and medulloblastomas. Finally, the role of mutations in epigenetic regulators is a known phenomenon in medulloblastomas [48]. In contrast, these events are still poorly characterized in PATs or MNTIs [8,9]. The contribution of these mediators to the divergent pathobiology between these three entities remains elusive, and further studies are needed to support these hypotheses.

**Table 2:** Differentially methylated genes and their relationship with MNTI and PAT.

Gene Name	Gene Function Description	Status MNTI-PAT vs. MBG3	References
*ACOT7*	Lipid metabolism	Hypermethylated	[49]
*ACSS3*	Lipid metabolism	Hypermethylated	[50]
*ANO4*	Ca2+ activated Cl- channels	Hypomethylated in PAT Hypermethylated in MNTI	[51]
*GLT8D2*	*GLT8D2* is a glycosyltransferase of apoB100 that regulates apoB100 levels ER	Hypomethylated	[52]
*HLA-J*	Major histocompatibility complex, class I, J (pseudogene)	Hypermethylated	[53]
*KIAA0040*	HLA-DR11-restricted T-cell epitope encoded by *KIAA0040*, alcohol dependency	Hypomethylated	[54]
*LIME1*	Adaptor protein involved in CD4 and CD8 coreceptor signaling	Hypermethylated	[55]
*OXGR1*	Receptor for alpha-ketoglutarate, expressed in adrenal glands. Frequently hypermethylated in hepatocellular carcinoma	Hypermethylated in PAT, Hypomethylated in MNTI	[56]
*PCDHGA4*	Neural cadherin-like cell adhesion genes, hypermethylated in neuroblastoma	Hypomethylated	[57,58]
*RIIAD1*	Regulatory subunit of type II PKA R-subunit domain containing 1	Hypermethylated	[59]
*SLC17A8*	GLUT3 (Slc17a8) is expressed in neurons classically defined by their use of another transmitter, such as acetylcholine and serotonin	Hypomethylated	[60]
*TMEM176B*	Innate immune checkpoint	Hypomethylated	[61]
*TRPC7*	Ca2+ signaling pathway	Hypomethylated	[62]
*ZNF154*	Candidate Tumor Suppressor *ZNF154* suppresses invasion and Metastasis in NPC by inhibiting the EMT via Wnt/β-catenin signaling	hypomethylated in PAT Hypermethylated in MNTI	[63]
*ACTA1*^†^	α-skeletal actin, *WNT* target	Hypomethylated in PAT Hypermethylated in MNTI	[64]
*ALDH3A1*^†^	Target of *WNT* pathway	Hypermethylated	[65]
*ASCL2*^†^	*SMYD3* controls a Wnt-responsive epigenetic switch for *ASCL2* activation and cancer stem cell maintenance Achaete-scute like 2 (*ASCL2*) is a target of Wnt signaling and is upregulated in intestinal neoplasia	Hypermethylated	[64,66,67,68]
*CCDC8*^†^	Wnt inhibition of *CCDC8* phosphorylation or patient-derived mutations	Hypomethylated	[69]
*ESRP2*^†^	Wnt pathway genes are enriched in genes downregulated by *ESRP2*	Hypomethylated	[70]
*RUNX3*^†^	*RUNX3* inhibits glioma survival and invasion via suppression of the β-catenin/TCF-4 signaling pathway	Hypermethylated	[71]
*ISLR2* *	Essential role of Linx/Islr2 in the development of the forebrain anterior commissure The LRR receptor Islr2 is required for retinal axon routing at the vertebrate optic chiasm	Hypermethylated in PAT, Hypomethylated in MNTI	[72,73]
*LYPD1* *	Synaptic signaling	Hypomethylated	[74]
*NARR/RAB34* *	Genomic characterization of Gli-activator targets in Sonic Hedgehog-mediated neural patterning	Hypermethylated in PAT, Hypomethylated in MNTI	[75]
*RFX4* *	Zebrafish Rfx4 controls dorsal and ventral midline formation in the neural tube	Hypermethylated in PAT, Hypomethylated in MNTI	[76]
*SSH2* *	Regulation of actin filaments; neural development and function	Hypomethylated	[77]
*TUBA1C* *	Down-regulation of *TUBA1C* significantly reduces proliferation and migration in HCC cells Transcriptome analysis of adherens junction pathway-related genes after peripheral nerve injury	Hypomethylated	[78,79]
*VRK2* *	*VRK2A* is an A-type lamin-dependent nuclear envelope kinase that phosphorylates BAF. Associated with schizophrenia and epilepsy	Hypomethylated	[80,81]
*ALX4* *^†^	*ALX4*, an epigenetically down regulated tumor suppressor, inhibits breast cancer progression by interfering Wnt/β-catenin pathway *ALX4* relays sequential FGF signaling to induce lacrimal gland morphogenesis *ALX4* and *MSX2* play phenotypically similar and additive roles in skull vault differentiation *ALX4*, a transcriptional activator whose expression is restricted to sites of epithelial-mesenchymal interactions	Hypermethylated	[82]
*MEIS1* *^†^	Cardiac regeneration, stem cells and cancer Meis1 coordinates cerebellar granule cell development by regulating Pax6 transcription, BMP signaling, and Atoh1 degradation. Meis1 is specifically expressed by Sox2(+) stem cells, which give rise to all dental epithelial cell lineages Differential transcriptional regulation of meis1 by Gfi1b and its cofactors LSD1 and CoREST. Potential RA downstream targets play a crucial role in normal development; Combined overexpression of Hoxa9 and Meis1 in hematopoietic stem and progenitor cells (HSPCs) in mice leads to AML and an associated increase in the level of unphosphorylated (“activated”) β-catenin; WNT/β-catenin pathway activation in Myc immortalized cerebellar progenitor cells inhibits neuronal differentiation and generates tumors resembling medulloblastoma (whereas ptf1a, Meis1 and Lhx2 were used as markers of cells in the cerebellar VZ; Meis1 expression was seen in both WNT and Shh groups)	Hypomethylated	[40]
*MIR96* *^†^	Regulates *GFI1 MIR-96* is required for normal development of the auditory hindbrain. *MIR-96* was identified to indirectly regulate the Wnt/β-catenin pathway by suppressing HMG-box transcription factor protein 1 (HBP-1)	Hypermethylated	[83]

** * Genes involved in neural function/development. ^†^ Genes involved in WNT pathway.

## 4. Materials and Methods

### 4.1. Patient Population

Based on the methylation profile of a PAT (index case) showing morphologic overlap with a MNTI (from Sapienza University of Rome), institutional and consultation files from 3 large pediatric institutions (The Children’s Hospital of Philadelphia, Philadelphia, PA, United States; Ospedale Bambino Gesù, Roma, Italy; Università di Padova, Padua, Italy) were queried. Formalin-fixed, paraffin-embedded (FFPE) tumor samples with a morphologic diagnosis of MNTI (age 3 to 48 months) were retrieved following institutional review board approval. The pertinent clinical information was obtained from electronic medical records and available consultation material. A cohort of 36 previously published medulloblastomas diagnosed at Ospedale Bambino Gesù, Rome, Italy [17], was used as control group. This study was reviewed and approved by Bambino Gesù Children’s Hospital Ethical Committee (Protocol n° 1556-OPBG, 26th October 2018, final approval 15th January 2019).

### 4.2. Histology and Immunohistochemistry

Hematoxylin-eosin-stained sections and available immunohistochemical material were retrieved. The slides were reviewed by at least 3 pediatric pathologists. The mitotic count was determined by identification of the number of mitotic figures (cell divisions) in 10 high-power (400× magnification) fields. Additional immunohistochemical and molecular studies were conducted as needed. Immunohistochemistry for Phox2b (H-20, Santa Cruz Biotechnology, Dallas, TX, USA) was conducted in all cases.

### 4.3. Methylation Studies

DNA methylation profiling was performed as previously described [17,84], following protocols approved by the institutional review board. Tumor areas with highest tumor cell content (≥ 70%) were selected for analysis. DNA was extracted according to MagPurix FFPE DNA Extraction Kit (Resnova, Rome, Italy) for automatic extraction of genomic DNA. Samples were analyzed using Illumina Infinium Human Methylation EPIC BeadChip (EPIC) arrays (Illumina, San Diego, CA, USA), according to the manufacturer’s instructions, on the Illumina iScan Platform (Illumina, San Diego, CA, USA). In detail, 250 ng DNA was used as input material. Generated methylation data were compared to brain tumor classifier v11b4 [14], sarcoma classifier v12.2, and medulloblastoma classifier group 3/4 classifier v1.0, developed by Heidelberg University and DKFZ (https://www.molecularneuropatholog.org/mnp/classifier/all (accessed on 5 February 2021)) to assign a subgroup score for the tumors in the recognized methylation classes. EPIC BeadChip data were analyzed as previously reported [17,85] by means of R (V.3.6.1), using ChAMP package (V.2.16.1) for quality checks and filters to calculate methylation levels and functionally annotate probes at the gene level. Multidimensional scaling (MDS) on the cohort samples was performed using the cmdscale function with the Euclidean distance. A heatmap depicting normalized beta values was created by means of the pheatmap function, using the complete linkage method and Euclidean distance to cluster samples and probes. Low-quality CpG islands among the 48 islands previously identified [18] were removed from this clustering analysis. Differentially methylated regions (DMR) were identified by means of the Bumphunter tool (as implemented in ChAMP pipeline), setting the adjusted *p*-value threshold at 0.001 [86]. Functional enrichment analysis of DMRs was carried out by means of the WebGestalt tool [87], using overrepresentation analysis (ORA) on the Reactome and wikipathway cancer databases.

## 5. Conclusions

In summary, our study provides evidence that MNTI consistently shares a homogenous methylation profile with MB G3, and possibly with PAT. The morphologic and molecular overlap of these entities may represent a diagnostic pitfall in the routine diagnostic workup of biopsy material. Thus, further correlation with imaging and clinical features may prevent a potential misdiagnosis. A comprehensive methylation profiling supports our results and, for the first time, confirms the notion of a histogenetic relationship between these three tumors. However, our study also has several limitations, including a cohort with a relatively small number of cases (primarily due to these tumors’ rare nature) and its retrospective format (facilitating selection bias and incomplete or lost follow-up information). Besides, we did not conduct gene expression analysis or identification of single nucleotide variants in epigenetic modifiers in our cohort. The possible role of a multipotent progenitor cell (derived from early migratory cranial neural crest cells) in the histogenesis of these tumors and the influence of the anatomical site, tumor microenvironment, and additional cytogenetic events in their divergent biologic behavior deserve further investigation.

## Figures and Tables

**Table 1:** Clinicopathological and molecular features of seven MNTIs and one PAT.

Case No.	Sex	Age (mo)	Size (cm)	Location	Morphological Diagnosis	Idat	Brain Tumor Classifier Result	Calibrated Scores	MB G3/G4 Classifier	Calibrated Scores
1	M	12	N/A	Pineal	PAT	200992280039_R07	PLEX PED B	0.63	Subtype III	0.99
2	M		N/A	Pineal	PAT (relapse)	200992280039_R08	MB, G3	0.98	Subtype II /III	0.45/0.4
3	M	48	2	Neck	MNTI	203939360062_R07	MB, G3	0.99	Subtype II	0.99
4	F	3	4	Left maxilla	MNTI	203724130138_R05	MB, G3	0.98	Subtype II	0.99
5	M	7	3.5	Left maxilla	MNTI	203724130138_R06	PLEX PED B/MB, G3	0.38/0.27	Subtype II/III	0.48/0.31
6	M	4	4.3	Left maxilla	MNTI	203724130138_R07	MB, G3	0.95	Subtype II	0.85
7	M	9	2.5	Right paratesticular	MNTI	203939360072_R02	PLEX PED B	0.29	Subtype II	0.64
8	M	4	4.5	Right maxilla	MNTI	203939360072_R03	MB, G3	0.97	Subtype II	0.72
9	F	8	3	Left hard palate	MNTI	203939360072_R04	MB, G3	0.93	Subtype II	0.72

Legend: PLEX PED B = Methylation class plexus tumor, subclass pediatric B. MB, G3 = Methylation class medulloblastoma, subclass group 3. PAT = Pineal anlage tumor. MNTI = Melanotic neuroectodermal tumor of infancy.

## Data Availability

The data presented in this study are available in the provided Supplementary Materials.

## Supplementary Materials

The following are available online at https://www.mdpi.com/2072-6694/13/4/706/s1, Figure S1: Box-and-whisker plots depicting the maximum raw classification scores (highlighted in red) of the indicated tumor samples (red dot) in the first methylation class of the brain tumor classifier assigned to each sample. Grey dots represent the reference cases in the methylation class, Figure S2: Cluster dendrogram with bootstrap analysis (1000 iterations) values on the 39 high-quality CpG islands in the Hovestadt set (Hovestadt et al., 2013) for the samples in the cohort. The bootstrap probability (BP) values are depicted in green, the approximately unbiased (AU) probability values (p-values) are reported in red. p-value of a cluster is a value between 0 and 1, which indicates how strongly the cluster is supported by data. Values are expressed in percentage. Edge numbers are in grey. Clusters with AU larger than 95% are highlighted by red boxes. Progonoma (MNTI/PA); Medulloblastoma (MB), Figure S3: Copy number variation (CNV) profile analysis of the indicated PAT/MNTI. Depiction of structural rearrangements involving autosomes and X/Y chromosome. Gains/amplifications represent positive (green) and losses represent negative (red) deviations from the baseline. Twenty-nine tumor relevant genomic regions are highlighted, Table S1: Annotated probes located in differentially methylated regions (DMRs, p-value < 0.001) between MNTI/PAT and Group 3, Table S2: Differentially methylated regions (DMRs, p-value < 0.001) between MNTI/PAT and Group 3. Top 30 DMRs used for functional enrichment analyses are highlighted in blue, Table S3: Methylation level differences in probes from significant DMRs in MNTI/PAT (blue) vs. MBG3 (black), Table S4: Webgestalt analysis, from total DMR genes Supplementary information (zip file)
