# Supplementary material for: Melanotic Neuroectodermal Tumor of Infancy (MNTI) and Pineal Anlage Tumor (PAT) Harbor A Medulloblastoma Signature by DNA Methylation Profiling
Source: Cancers (Basel). 2021 Feb 9;13(4):706. doi: 10.3390/cancers13040706 (PMC7916108; doi:10.3390/cancers13040706)
Supplement: Supplementary file 1 [file cancers-13-00706-s001.zip › Supplementary information/MNTI PAT MBG4_G4 C reports/MNTI1 _ idat_reportMeduloblastoma_v1_0_version_1_0_203939360062_R07C01.pdf]

Methylation profiling report

General information

Sentrix ID:	**203939360062_R07C01**
Array type:	**EPIC**
Material type:	**FFPE DNA**
Gender:	**male**

Meduloblastoma methylation classifier results (v1.0)

Methylation classes (MCs with score >= 0.3)	Calibrated score	Interpretation	
medulloblastoma, subclass group 3/4, subtype II	0.99	match	✔

Legend: ✔ Match (score >= 0.9) ✖ No match (score < 0.9): possibly still relevant for low tumor content and low DNA quality cases. ● Match to MC family member (score >= 0.5)

Class descriptions

Medulloblastoma, subclass group 3/4, subtype II: The methylation class "medulloblastoma, subclass group 3/4, subtype II" is comprised of tumors with the diagnosis medulloblastoma. Histologically, most cases fall into the classic (50%) or large-cell/anaplastic group (45%) and 5% cases fall in the DN/MBEN group. Median age is 5 years (range 1 to 17). The majority (55%) are M+. Male:Female ratio is 3.3:1. Subtype II medulloblastomas are enriched for chr8 and chr17, isochromosome 17q (i17q) , chr13q gain and chr 16 loss, and are enriched for MYC amplification (20% of cases). Subtype% in II is high-risk (5-year OS ~ 49% in retrospective series). This subtype is comprised of 100% Grp3 subgroup tumors.

Copy number variation profile

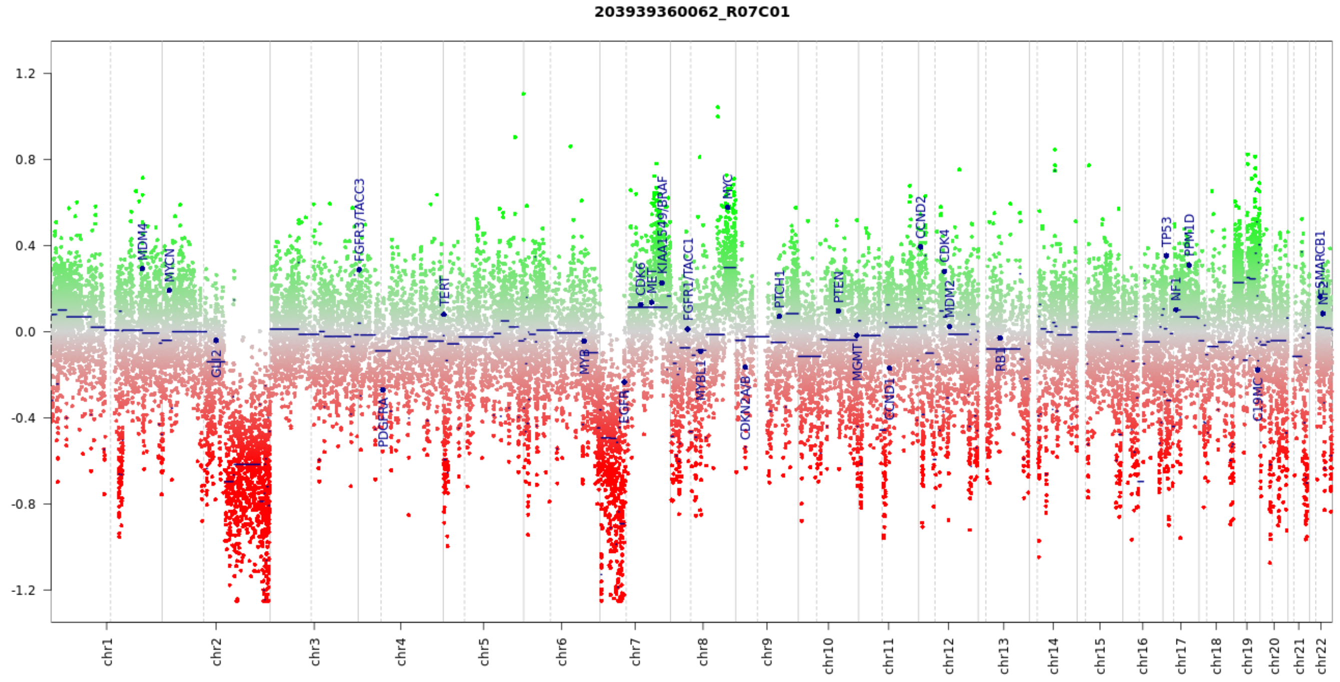

Depiction of chromosome 1 to 22 (and X/Y if automatic prediction was successful). Gains/amplifications represent positive, losses negative deviations from the baseline. 29 brain tumor relevant gene regions are highlighted for easier assessment.
(see Hovestadt & Zapatka, http://www.bioconductor.org/packages/devel/bioc/html/conumee.html)

MGMT promotor methylation (MGMT-STP27)

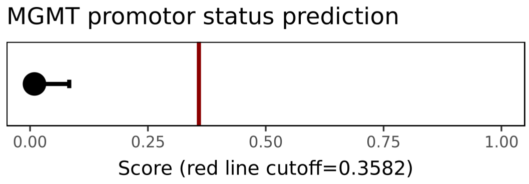

Status	Estimated	CI lower	CI upper
unmethylated	0.00932	9.8E-4	0.08311

(see Bady et al, J Mol Diagn 2016; 18(3):350-61)

Disclaimer

Classification using methylation profiling is a research tool under development, it is not verified and has not been clinically validated. Implementation of the results in a clinical setting is in the sole responsibility of the treating physician.
Intended for non-commercial use only.

Run information

Report: idat_reportMeduloblastoma_v1_0 Version 1.0
Task version:

Task	Version
idat_qc	2.0
idat_rs_gender	2.0
idat_predictMGMT	2.0
idat_cnvp	3.0
idat_predictMeduloblastoma	1.0
