# Supplementary material for: Melanotic Neuroectodermal Tumor of Infancy (MNTI) and Pineal Anlage Tumor (PAT) Harbor A Medulloblastoma Signature by DNA Methylation Profiling
Source: Cancers (Basel). 2021 Feb 9;13(4):706. doi: 10.3390/cancers13040706 (PMC7916108; doi:10.3390/cancers13040706)
Supplement: Supplementary file 1 [file cancers-13-00706-s001.zip › Supplementary information/MNTI PAT BTC reports/MNTI4 _ idat_reportBrain_v11b4_version_2_0_203724130138_R07C01.pdf]

Methylation profiling report

GERMAN CANCER RESEARCH CENTER IN THE HELMHOLTZ ASSOCIATION
Heidelberg University Hospital
MolecularNeuropathology.org

General information

Sentrix ID:	**203724130138_R07C01**
Array type:	**EPIC**
Material type:	**FFPE DNA**
Gender:	**male**

Brain tumor methylation classifier results (v11b4)

Methylation classes (MCs with score >= 0.3)	Calibrated score	Interpretation	
methylation class family Medulloblastoma group 3 and 4	0.96	match	✔
**MC family members with score >= 0.1**			
methylation class medulloblastoma, subclass group 3	0.95	match	●

Legend: ✔ Match (score >= 0.9) ✘ No match (score < 0.9): possibly still relevant for low tumor content and low DNA quality cases. ● Match to MC family member (score >= 0.5)

Class descriptions

Methylation class family Medulloblastoma group 3 and 4: The methylation class family "Medulloblastoma class 3 and 4" comprises the methylation classes medulloblastoma, class 3 and medulloblastoma, class 4.

Methylation class medulloblastoma, subclass group 3: The methylation class "medulloblastoma, subclass group 3" is comprised of tumors with the diagnosis medulloblastoma, genetically defined, group 3. Histologically most cases fall into the classical and large cell/anaplastic groups. Tumors are located in the cerebellum, typically in the vermis. Median age is 4 years (range 1 to 17). Group 3 medulloblastomas are more common in males than females. MYC amplification, aneuploidy, isochromosome 17q and GFI1/1B activation by enhancer hijacking are recurrent features, but a fraction lack an obvious driving genetic change.

Copy number variation profile

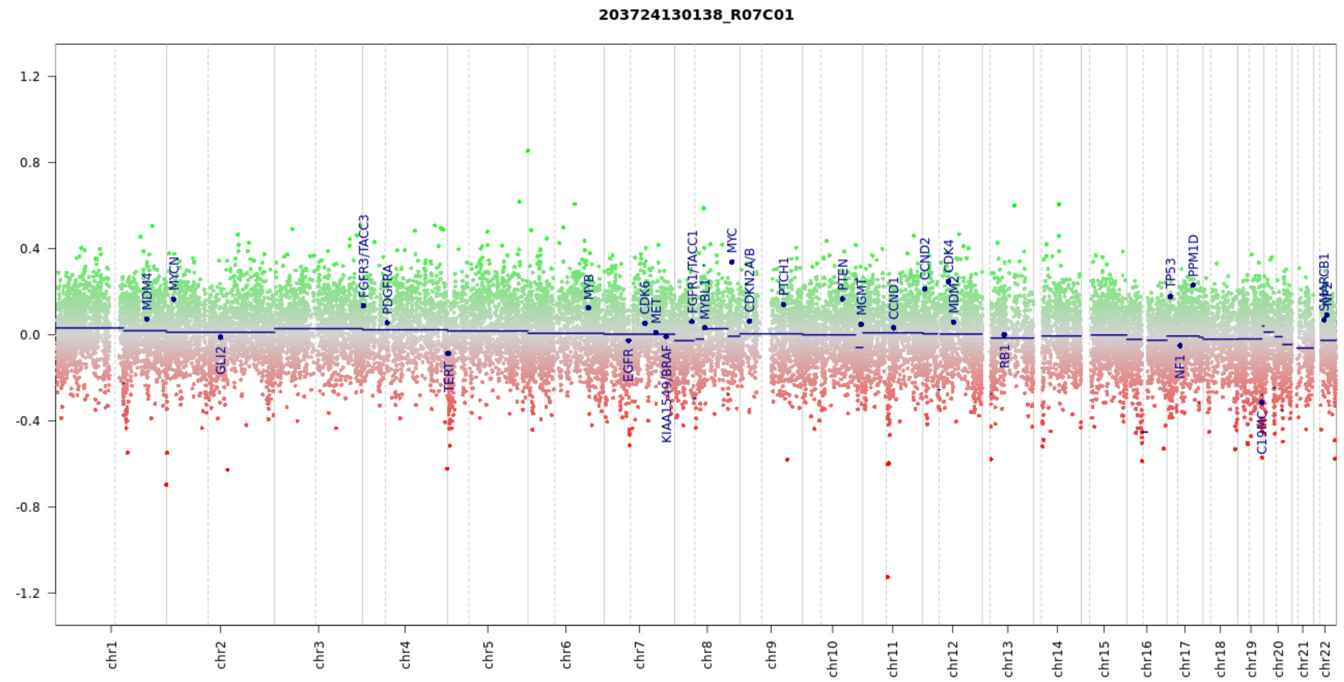

Depiction of chromosome 1 to 22 (and X/Y if automatic prediction was successful). Gains/amplifications represent positive, losses negative deviations from the baseline. 29 brain tumor relevant gene regions are highlighted for easier assessment.
(see Hovestadt & Zapatka, http://www.bioconductor.org/packages/devel/bioc/html/conumee.html)

MGMT promotor methylation (MGMT-STP27)

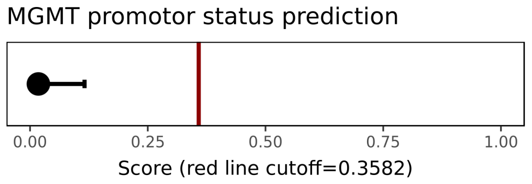

Status	Estimated	CI lower	CI upper
unmethylated	0.01773	0.00248	0.11572

(see Bady et al, J Mol Diagn 2016; 18(3):350-61)

Disclaimer

Classification using methylation profiling is a research tool under development, it is not verified and has not been clinically validated. Implementation of the results in a clinical setting is in the sole responsibility of the treating physician.
Intended for non-commercial use only.

Run information

Report: idat_reportBrain_v11b4 Version 2.0
Task version:

Task	Version
idat_qc	2.0
idat_predictBrain	2.1
idat_rs_gender	2.0
idat_predictMGMT	2.0
idat_cnvp	3.0
