# Supplementary material for: Melanotic Neuroectodermal Tumor of Infancy (MNTI) and Pineal Anlage Tumor (PAT) Harbor A Medulloblastoma Signature by DNA Methylation Profiling
Source: Cancers (Basel). 2021 Feb 9;13(4):706. doi: 10.3390/cancers13040706 (PMC7916108; doi:10.3390/cancers13040706)
Supplement: Supplementary file 1 [file cancers-13-00706-s001.zip › Supplementary information/MNTI PAT BTC reports/MNTI5 _ idat_reportBrain_v11b4_version_2_0_203939360072_R02C01.pdf]

Methylation profiling report

General information

Sentrix ID:	**203939360072_R02C01**
Array type:	**EPIC**
Material type:	**FFPE DNA**
Gender:	**male**

Brain tumor methylation classifier results (v11b4)

Methylation classes (MCs with score >= 0.3)	Calibrated score	Interpretation	
methylation class family Plexus Tumor	0.45	no match	✗
**MC family members with score >= 0.1**			
methylation class plexus tumor, subclass pediatric B	0.29		
methylation class plexus tumor, subclass pediatric A	0.15		

Legend: ✓ Match (score >= 0.9) ✗ No match (score < 0.9): possibly still relevant for low tumor content and low DNA quality cases. ● Match to MC family member (score >= 0.5)

Class descriptions

Methylation class family Plexus Tumor: The methylation class family "Plexus Tumor" comprises the methylation classes plexus tumor, pediatric subtype A, plexus tumor, pediatric subtype B and plexus tumor, adult subtype.

Methylation class plexus tumor, subclass pediatric B: The methylation class "plexus tumor, subclass pediatric B" mainly comprises cases diagnosed as choroid plexus carcinomas (60%) but also atypical plexus papillomas (30%) and rarely classical plexus papillomas (10%). Over 95% of cases are located in the lateral ventricle (supratentorial). Most cases occur in young children with exceptional cases in adults; median age is 1 year (range 0 to 30). Numeric whole chromosome changes are frequent in this class, often including loss of chromosome 3, 5, 6, 11, 16, 17, 18, 19 and 22 and gain of chromosome 12, 14 and 20. The methylation class is closely related to methylation cluster 3 described in Thomas et al., Neuro Oncol. 2016.

Methylation class plexus tumor, subclass pediatric A: The methylation class "plexus tumor, subclass pediatric A" comprises cases diagnosed as choroid plexus papillomas and atypical choroid plexus papillomas. These tumors occur preferentially supratentorial in the lateral or 3rd ventricle but also infratentorial in or around the 4th ventricle; median age is 0 years (range 0 to 9). Additional characteristic molecular features of this class are not known to date. Numeric whole chromosome changes are frequent in this class, often including gain of chromosome 5, 8, 9, 11, 12, 14, 15, 20 and X (each in over 40% of cases).

Copy number variation profile

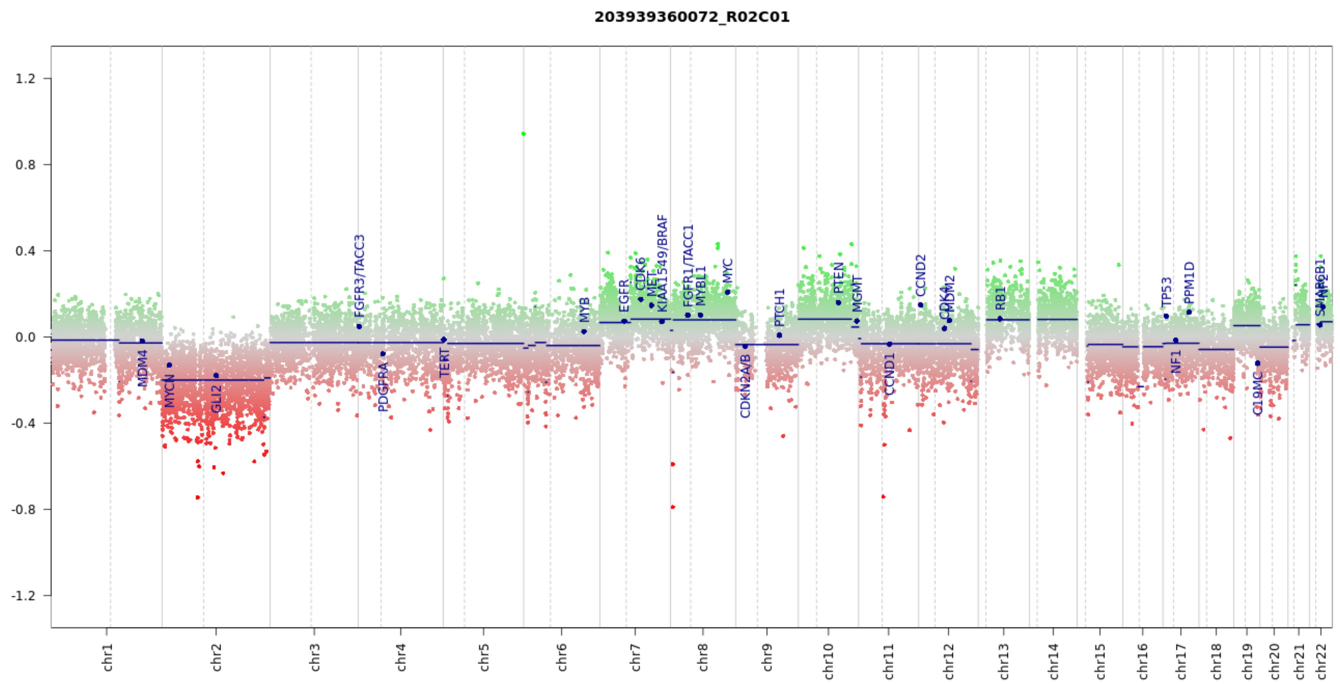

Depiction of chromosome 1 to 22 (and X/Y if automatic prediction was successful). Gains/amplifications represent positive, losses negative deviations from the baseline. 29 brain tumor relevant gene regions are highlighted for easier assessment.
(see Hovestadt & Zapatka, http://www.bioconductor.org/packages/devel/bioc/html/conumee.html)

MGMT promotor methylation (MGMT-STP27)

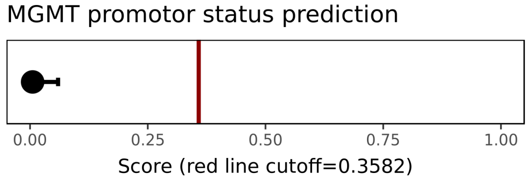

Status	Estimated	CI lower	CI upper
unmethylated	0.00545	4.7E-4	0.05952

(see Bady et al, J Mol Diagn 2016; 18(3):350-61)

Disclaimer

Classification using methylation profiling is a research tool under development, it is not verified and has not been clinically validated. Implementation of the results in a clinical setting is in the sole responsibility of the treating physician.
Intended for non-commercial use only.

Run information

Report: idat_reportBrain_v11b4 Version 2.0
Task version:

Task	Version
idat_qc	2.0
idat_predictBrain	2.1
idat_rs_gender	2.0
idat_predictMGMT	2.0
idat_cnvp	3.0
