# Supplementary figures and images for: Melanotic Neuroectodermal Tumor of Infancy (MNTI) and Pineal Anlage Tumor (PAT) Harbor A Medulloblastoma Signature by DNA Methylation Profiling
Source: Cancers (Basel). 2021 Feb 9;13(4):706. doi: 10.3390/cancers13040706 (PMC7916108; doi:10.3390/cancers13040706)

# PAT

PLEX, PED B

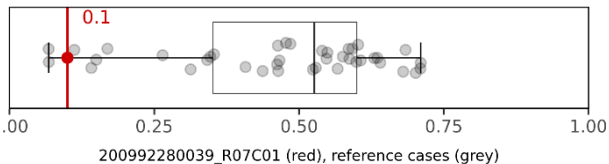

# PATr

MB, G3

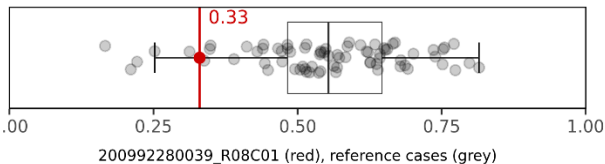

# MNTI1

MB, G3

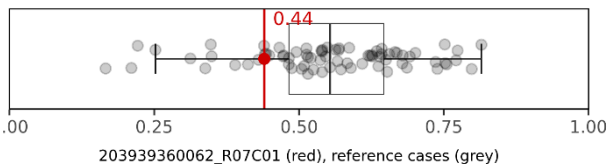

# MNTI2

MB, G3

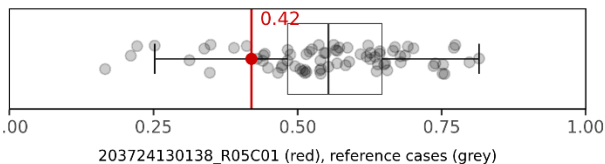

# MNTI3

MB, G3

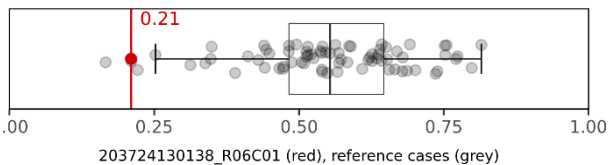

# MNTI4

MB, G3

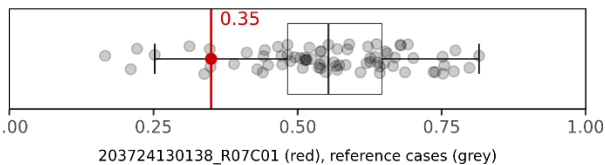

# MNTI5

MB, G3

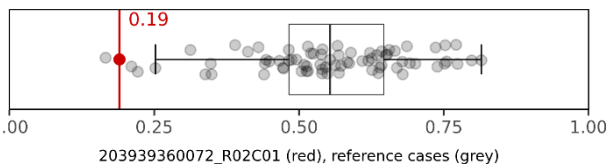

# MNTI6

MB, G3

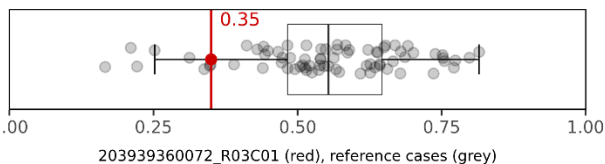

# MNTI7

MB, G3

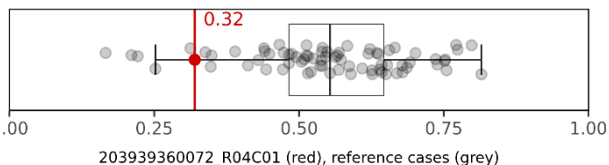

Supplement: Supplementary file 1 [file cancers-13-00706-s001.zip › Supplementary Figure 1.pdf]

# Cluster dendrogram with p-values (%)

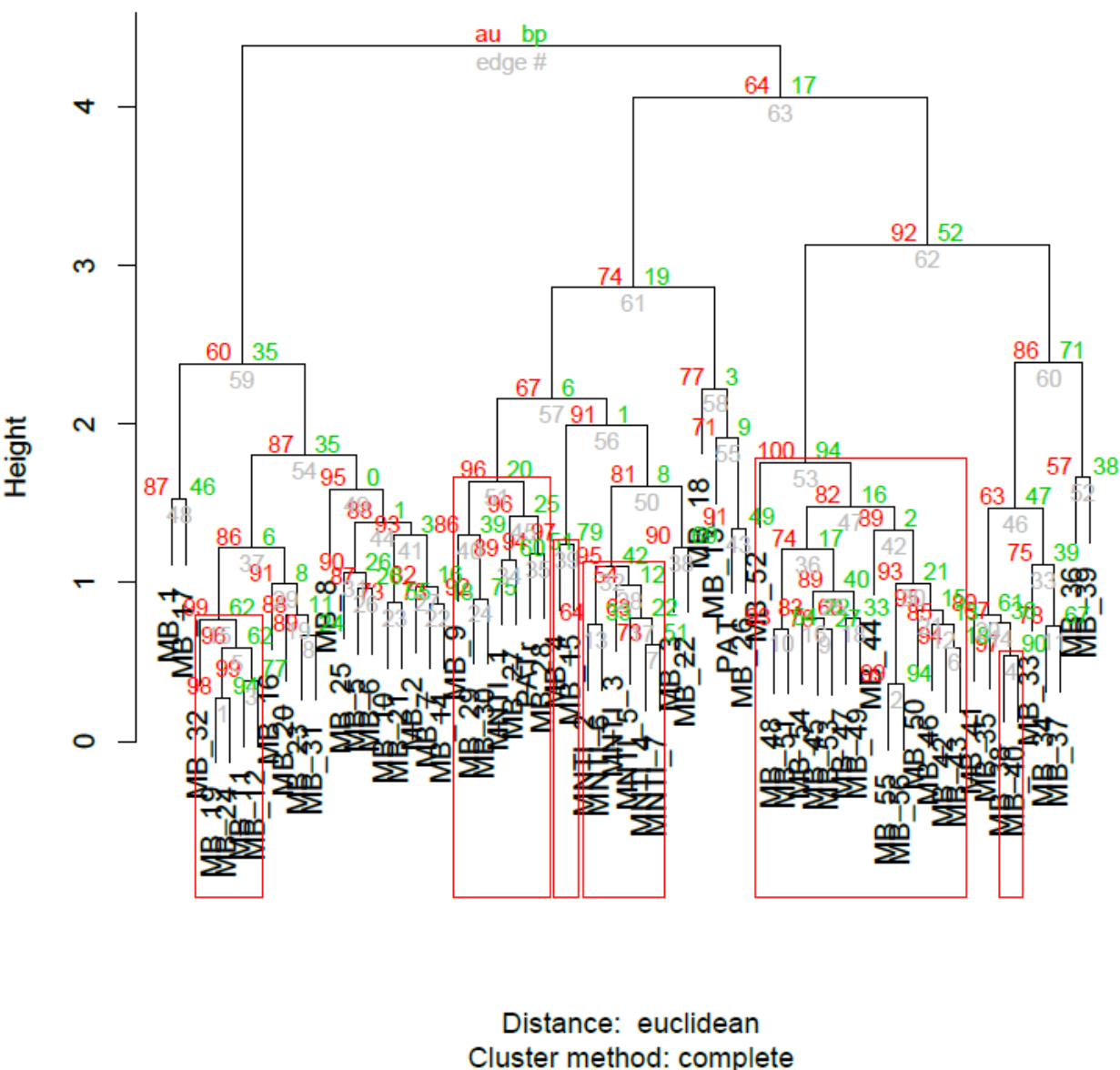

Supplement: Supplementary file 1 [file cancers-13-00706-s001.zip › Supplementary Figure 2.pdf]

# PAT

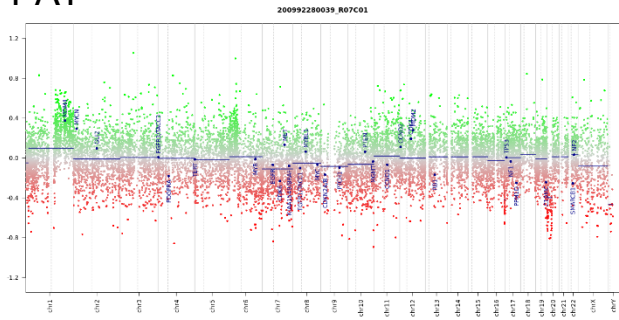

# PATr

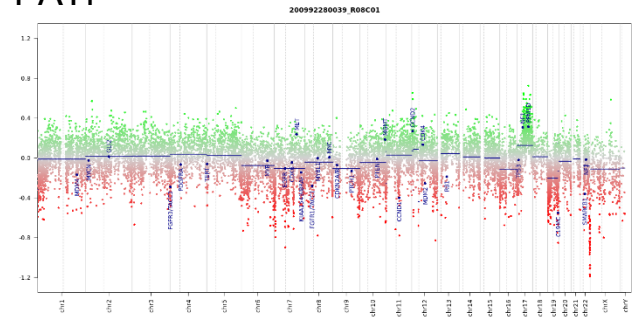

# MNTI1

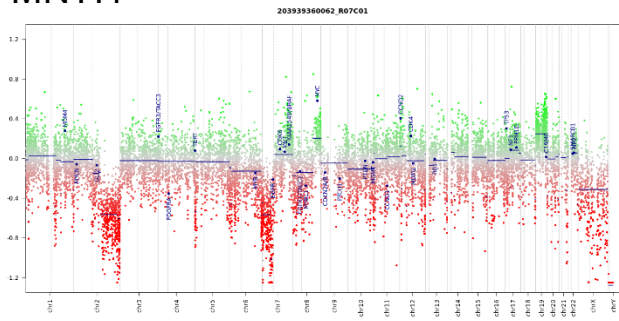

# MNTI2

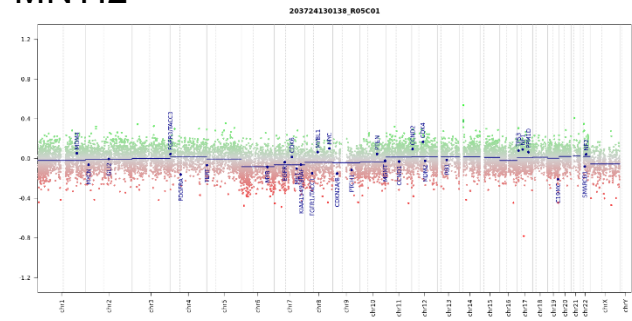

# MNTI

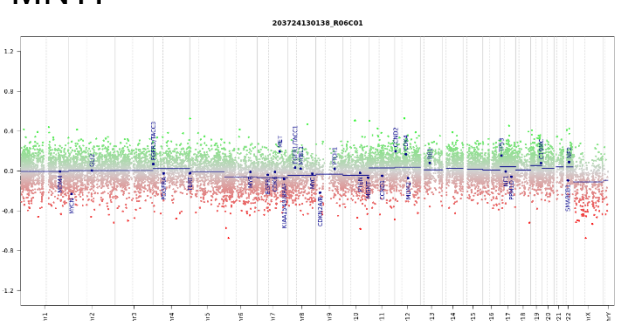

# MNTI4

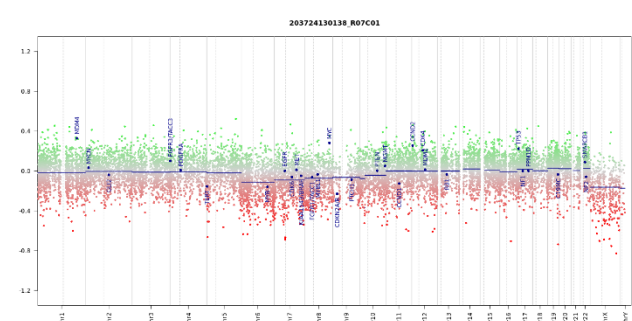

# MNTI5

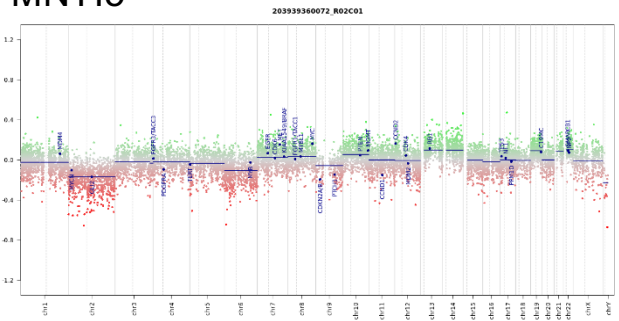

# MNTI6

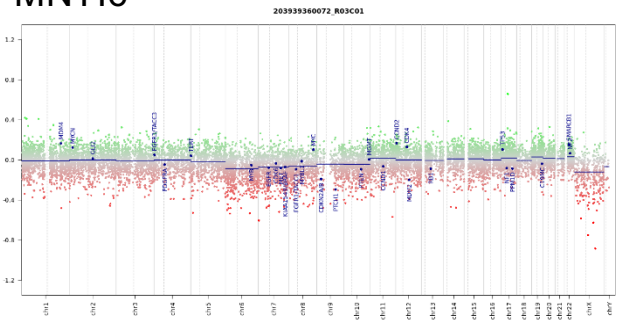

# MNTI7

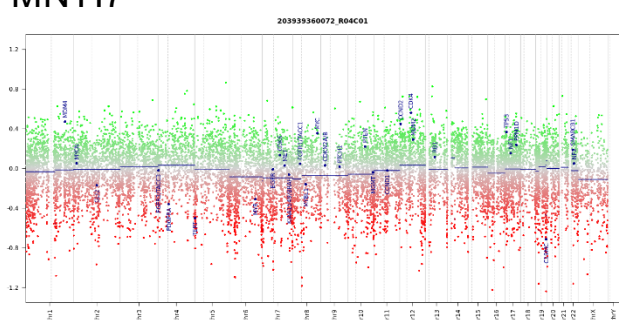

Supplement: Supplementary file 1 [file cancers-13-00706-s001.zip › Supplementary Figure 3.pdf]
